# Efficient and Consistent Orthotopic Osteosarcoma Model by Cell Sheet Transplantation in the Nude Mice for Drug Testing

**DOI:** 10.3389/fbioe.2021.690409

**Published:** 2021-09-24

**Authors:** Hongwei Wu, Zhengxi He, Xianan Li, Xuezheng Xu, Wu Zhong, Jie Bu, Gang Huang

**Affiliations:** ^1^ Department of Orthopedics, Hunan Cancer Hospital and The Affiliated Cancer Hospital of Xiangya School of Medicine, Central South University, Changsha, China; ^2^ Department of Oncology, Xiangya Hospital, Central South University, Changsha, China; ^3^ Cancer Research Institute, Basic School of Medicine, Central South University, Changsha, China

**Keywords:** osteosarcoma, mice model, cell sheet, orthotopic transplantation, drug testing

## Abstract

Osteosarcoma is a big challenge on clinical treatment. The breakthrough associated with osteosarcoma in basic research and translational research depends on the reliable establishment of an animal model, whereby mice are frequently used. However, a traditional animal modeling technique like tumor cell suspension injection causes batch dynamics and large mice consumption. Here, we suggested a novel approach in establishing an orthotropic osteosarcoma model in nude mice rapidly by cell sheet culture and transplantation. Our findings demonstrated that the 143b osteosarcoma cell sheet orthotopically implanted into the nude mice could form a visible mass within 10 days, whereas it took over 15 days for a similar amount of cell suspension injection to form a visible tumor mass. Living animal imaging results showed that a tumor formation rate was 100% in the cell sheet implantation group, while it was 67% in the cell suspension injection group. The formed tumor masses were highly consistent in both growth rate and tumor size. Massive bone destruction and soft tissue mass formation were observed from the micro CT analysis, suggesting the presence of osteosarcoma. The histopathological analysis demonstrated that the orthotropic osteosarcoma model mimicked the tumor bone growth, bone destruction, and the lung metastasis. These findings imply that such a cell sheet technology could be an appropriate approach to rapidly establish a sustainable orthotropic osteosarcoma model for tumor research and reduce mice consumption.

## Introduction

Osteosarcoma (OS) is the most common bone tumor in adolescents. The fatality rate of osteosarcoma patients is high since 50% of them later develop tumor metastasis (Ellegast et al., 2011; Kleinerman, 2016; Kovar, 2018). As such, it is necessary to establish a practical animal model to study the OS pathogenesis and to explore the potential prognosis (Geller et al., 2015; Pavlou et al., 2019).

The construction of a mouse OS model is the most cost-effective approach to simulate the human body environment and hence study the development of human OS (Kansara et al., 2014; Yu et al., 2021; Xiaobin et al., 2021; Wilk and Zabielska-Koczywas, 2021). Methods to induce a tumor model in mice include spontaneous tumorigenesis, carcinogen induction, transgenic mouse models (Lahr et al., 2021; Nevil et al., 2021), and human patient-derived xenograft models (Wu et al., 2021). The OS model consists of subcutaneous tumorigenesis and *in situ* OS model. The *in situ* OS model can simulate the OS biological process occurrence and progression, tumor invasion, and metastasis. The traditional OS *in situ* model entails cell suspension injection. It is easy to operate and learn. However, it is associated with limitations like suspension leakage, low survival rate, unstable tumor formation rate, and prolonged tumor formation duration, which often results in a large use of mice for the experiments.

To overcome that limitation, some researchers have used matrix glue or biomaterials as scaffolds to conduct local injection of tumor cells to accelerate tumor formation (Bhattacharjee et al., 2017; Pavlou et al., 2019). However, the existing scaffold materials exhibit defects like insufficient biological activity, unstable degradation, and possible immunogenicity in immunocompetent mice. This results in immune response and inflammation and potential to cause cell mortality (Yang et al., 2005). For the immunodeficient mice, despite the decreased inflammatory response, the scaffold might still affect cell growth and activities (Rodriguez et al., 2009; Bouvet-Gerbettaz et al., 2014). Recently, because of the development of tissue engineering and tumor organ technology, cell sheet technology has earned attention of researchers recently (Suzuki et al., 2014; Kobayashi et al., 2018; Akimoto et al., 2019; Lu et al., 2019).

Cell sheet techniques could be used to collect cells without using proteolytic enzymes like trypsin. As such, the cell–cell junction, extracellular matrix, and cell flake structure are preserved. This ensures a high cell density and even cell distribution. Besides, cell sheets prepared *via* intercellular junctions and extracellular matrix secretion are not affected by immune and inflammatory responses, tissue collapse, and tissue formation resulting from stent implantation (Bhattacharjee et al., 2017; Roseti et al., 2017; Yorukoglu et al., 2017; Li et al., 2019).

To the best of our knowledge, there is scanty research concerning the use of the cell sheets technique for *in situ* modeling of osteosarcoma. We hypothesized that osteosarcoma cell sheets could be used for efficient transplantation and tumorigenesis. We emphasized reduction in the number of experimental animals and standardizing the conditions for technological development.

## Materials and Methods

### Cell Culture and Cell Sheet Preparation

Luciferase-transfected 143b osteosarcoma cell line (Luc-143b, ATCC) was cultured in a 2D monolayer culture dish. The dish contained high-glucose DMEM (Invitrogen, CA, United States) culture medium comprising 10% fetal bovine serum (Gibco, Austria) supplemented with 1% penicillin/streptomycin. The medium was changed every 2 days. When the cells reached 95% confluence, they were digested with 0.5% trypsin. The cell suspension was seeded in a 12-well UPCELL culture plate (Thermofish, United States) at a density of 20 × 10^4^/well. On the fifth day, the cells reached 100% confluence. The culture medium was replaced with a cold DMEM. The culture plate was placed in the hood at room temperature for 5–10 min. The osteosarcoma cell sheet would then detach from the culture plate automatically. The cell sheets were then used in the subsequent experiments.

### Cell Sheet Histology and Immunocytochemistry

The harvested intact Luc-143b cell sheets were washed with PBS and then fixed with 4% paraformaldehyde for 20 min. Later, they were embedded into paraffin for histological slicing at 5 μm per slice. The resultant slices were stained with hematoxylin and eosin (H&E) for histological observation. Concerning collagen staining, the fixed cell sheet was blocked with 0.1% normal bovine serum albumin. It was then incubated with a 1:500 diluted Anti-Collagen I antibody (abcam, ab34710, United States) over night at 4°C. A 1:1,000 diluted second antibody conjugated with Alexa Fluor 488 (goat anti-rabbit 2nd antibody, ab150077) was incubated for 2 h at room temperature. The samples were imaged using an upright fluorescence microscope (Nikon, Japan).

### Osteosarcoma Cell Sheet Orthotopic Transplantation

**FIGURE 1:**
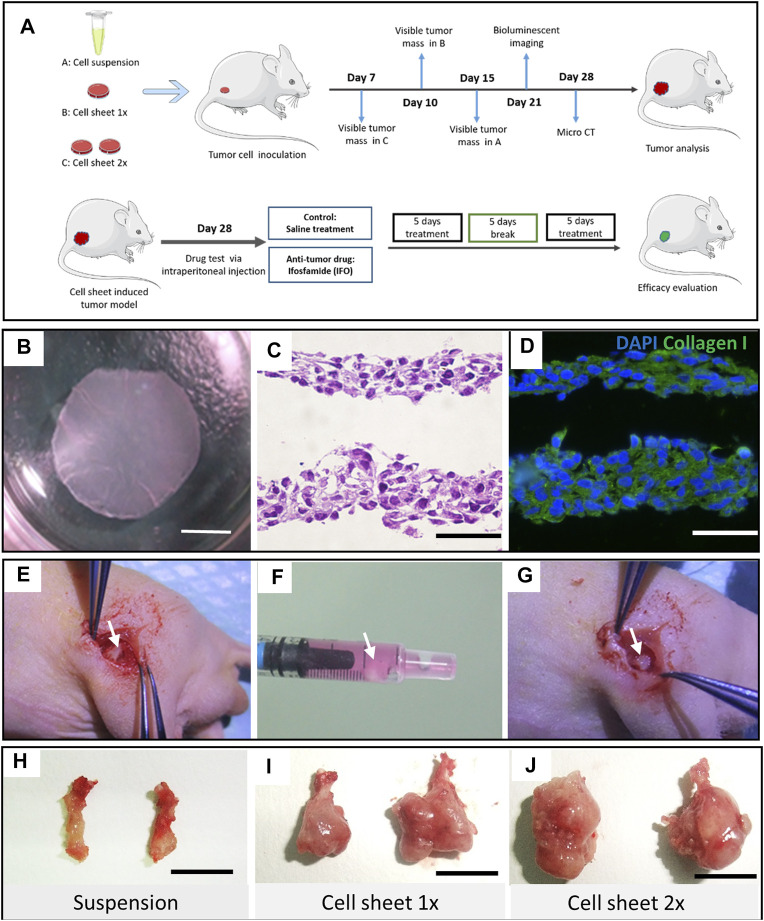
Study design and OS cell sheet characterization and implantation. **(A)** Schematic graph of the study design. Cell suspension, one piece of cell sheet and two pieces of cell sheet were transplanted into the distal femur of nude mice, respectively. **(B)** Intact Luc-143b OS cell sheet detached from a UP-cell 12-well culture well. Scar bar = 4 mm. **(C)** Hematoxylin and eosin staining of the cell sheet. Scar bar = 50 μm. **(D)** Immunofluorescence staining of collagen type I (green) and nuclear (blue) in the OS cell sheet. Scar bar = 50 μm. **(E)** An orthotropic distal femur defect was made. **(F)** OS cell sheet collected in the syringe. **(G)** OS cell sheet transplanted in site on the defect. Representative bone tumor lesion induced by cell suspension **(H)**, one piece of cell sheet **(I)**, and two pieces of cell sheet, **(J)** respectively, scale bar = 1 cm.

All experiments involving the use of animals in this study were approved by the Hunan Cancer Hospital Ethnic Committee on Animal Use (No. 2018-11). The schematic graph of this study is shown in Figure 1 A. To minimize the consumption of the mice, eighteen male BALB/c (nu/nu) nude mice were used for the first two batches of experiments to test efficient tumorigenesis *via* cell sheet transplantation. Four BALB/c nude mice were used for the third batch of experiment to validate the consistency of the cell sheet transplantation. Six-week-old male BALB/c nude mice weighing 20 g were anesthetized with phenobarbital (30 mg/kg) through intraperitoneal injection. A cylinder-shaped bone defect was created at the distal of lateral femoral condyle using a scalpel. One or two pieces of cell sheets were implanted respectively as experimental groups (Figures 1E–G). The same amount cell number of 100ul Luc-143b suspension was injected into the bone marrow cavity as a control. The mice were observed twice per week to assess mass formation. The longest diameter (*A*) and the shortest diameter (*B*) of the tumor were measured. The calculation formula of the tumor volume was Volume = (*A* × *B* ^2^)/2 (Yuan et al., 2009).

### Bioluminescent Imaging

On the 21st day after transplantation, the mice in all the groups were subjected to bioluminescent imaging using a bioluminescent imaging system (Perkin Elmer IVIS lumina LT Series III, MA, United States) for *in vivo* tumor detection. All the mice were anesthetized intraperitoneally using phenobarbital (30 mg/kg). Following a successful anesthesia, 100 μL fresh D-luciferin substrate (15 mg/ml, Solarbio, China) working solution was injected intraperitoneally. After 10 min, the nude mice were put into a live imaging system for image examination. The fluorescence range and tumor intensity were observed. The BL images were analyzed using Living Image 4.3 software (PerkinElmer).

### MicroCT Analysis

On the 28th day following transplantation, the mice in each group were euthanized. Then the femur samples were harvested and fixed with 4% paraformaldehyde for 24 h. After being subjected to alcohol immersion and sterilization, they were put into high-resolution *in vivo* x-ray microtomography (skyscan-1276, Bruker, Germany) for scanning. A three-dimensional image was made by neocon software. Bone destruction was analyzed using scan software. Graphical display and statistical analysis were conducted using Prism 8 (GraphPad Software, CA, United States).

### Histological Assessment and Immunohistochemistry Staining

All the samples were obtained and fixed in 4% paraformaldehyde for 24 h. They were later embedded in paraffin. Histology slices were made on a rocking microtome. Hematoxylin and eosin staining was performed to observe the pathological dynamics in masses. To detect the cell proliferation in the tumor tissue, the sections were incubated with the Ki-67 antibody (1:500, GB13030-2, Servicebio, China) and detected using goat anti-rabbit IgG H&L (HRP) antibody (1:200, GB23303, Servicebio, China). DAB (DAKO, K5007, Japan) was used as the chromogen and then the section was counterstained with hematoxylin. The positive percentage calculated by four pathological professionals without prior knowledge of the experimental process. To detect the fibroblasts infiltration and blood vessels in the tumor tissue, the sections were incubated with collagen 1A1 (1:1,000, GB11022, Servicebio, China) and α-SMA (1:500, GB111364, Servicebio, China) antibody. The following procedure was the same with Ki67 staining. To detect the cell origin in the tumor, the sections were stained with anti-HLA G antibody (1:200, ab7758, Abcam) and detected with goat anti-mouse IgG H&L Alexa Fluor 488 (ab150113).

### Antitumor Efficacy Test of Ifosfamide

To investigate the *in vivo* antitumor effect, the tumor-bearing mice were treated with ifosfamide (IFO, 0.1 mg/g) intraperitoneally 28 days after the tumor inoculation. Two cycles were conducted with 5 days a cycle (Figure 5A). Mesna (0.2 mg/g) was administrated intraperitoneally simultaneously to prevent the side effects of ifosfamide. The same amount of saline was administrated as the control. The administration was repeated for two cycles with an interval of 5 days. The mice were then euthanized, and tumor samples were harvested for the histology analysis. The tumor size of both groups was recorded at the end of the drug administration. The antitumor efficacy of IFO toward the tumor was estimated from the volume change of the tumor, which was calculated by the following formula: Tumor volume (*V*) = ab^2^/2, where “a” and “b” indicate the major and minor axes of the tumors, respectively. Immunostaining for TUNEL (GDP1041, Servicebio, China), Bcl-2 (1:200, GB12318, Servicebio, China), and caspase-3 (1:200, GB11383, Servicebio, China) was performed to detect the cell apoptosis. Chromogen and nucleic staining were the same as previously described in the histology staining section.

### Statistics

Data were presented as mean value with SD. Graphs and statistical analyses, using Student’s t-test, were performed with GraphPad Prism software (GraphPad Software, La Jolla, United States).

## Results

### Osteosarcoma Cell Sheet Formation and Orthotopic Transplantation

To culture an osteosarcoma cell sheet properly, our findings indicated that 50 × 10^3^ cells per well was the most appropriate initial cell density to form an intact cell sheet (Figure 1B). Either too high or too low cell densities resulted in failure of cell sheet formation or harvest (Supplementary Figures 1D–E). We compared the regular tissue culture plate with the UpCell culture plate in terms of the cell proliferation rate. There was no significant difference (*p* < 0.05) between the two groups (Supplementary Figure 1F). However, when comparing the 2D culture with 3D spheroid culture condition, the 3D culture condition exhibited a slower proliferation curve than the 2D culture condition (Supplementary Figure 1G–I). The H&E staining indicated that the cell sheet had a thin layer of 25–50 μm. Immunostaining results showed that collagen type I evenly distributed across the 143b cell sheet (Figures 1C,D).

To testify the feasibility of cell sheet orthotopic transplantation, we used a 1-ml syringe to transfer the cell sheet from the culture plate to the inoculation site (Figures 1E–G). We observed a tiny lesion 7 days after implantation in the two-piece cell sheet transplantation group. While in the suspension group, it was not observed until 15 days after suspension injection. Four weeks postimplantation, representative lesion samples in each group were displayed in which the two-piece sheet group had the largest tumor formation (Figures 1H–J).

### Tumor Formation and Consistency Analysis

**FIGURE 2:**
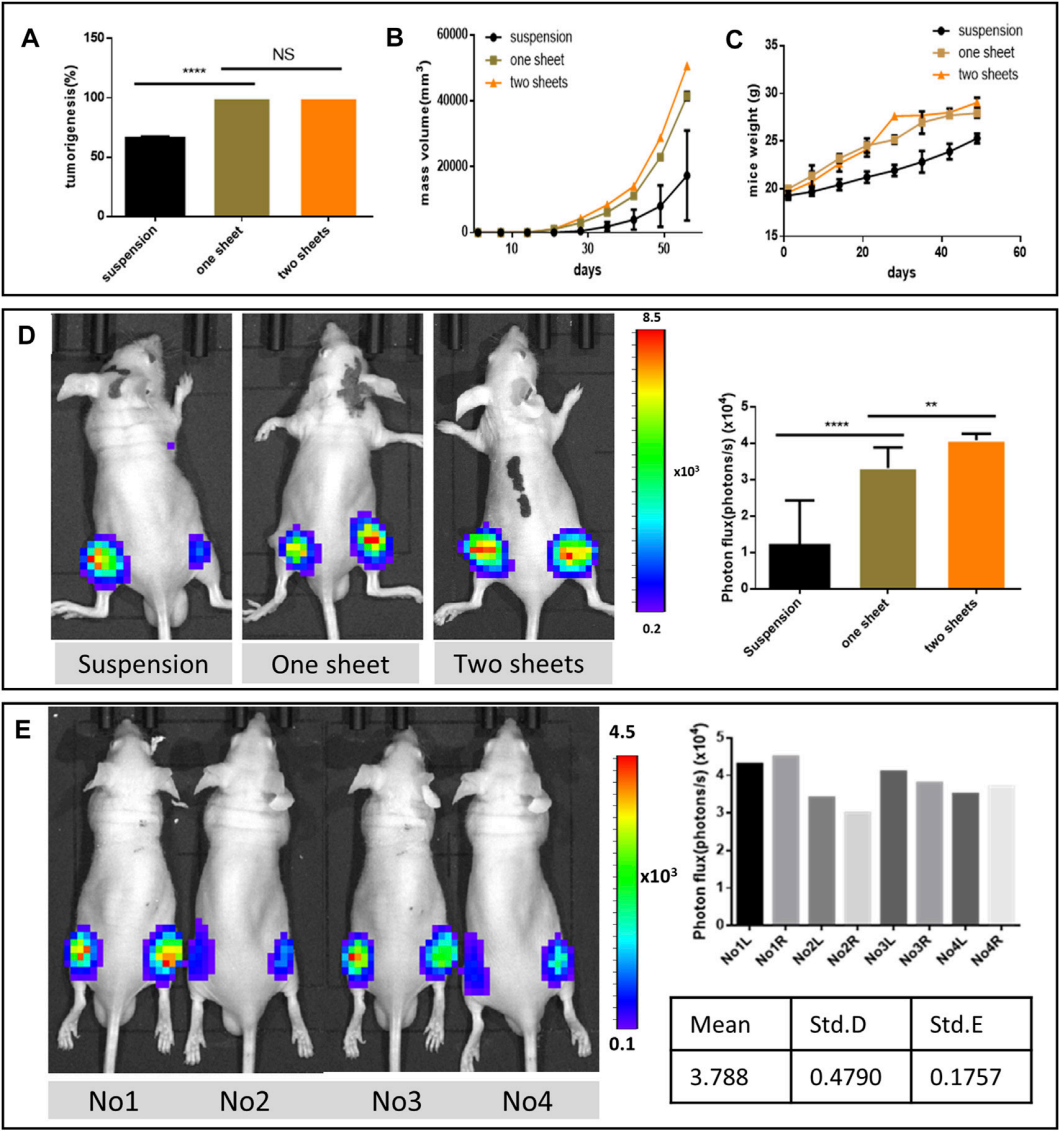
Tumor formation analysis. **(A)** Comparison of tumorigenesis rate. **(B)** Mass volume of the formed tumor. **(C)** Growth weight curve of the tumor formed mice. (n = 6, graph displays mean ± SEM). **(D)** Representative bioluminescent images of nude mice from different groups 21 days after orthotopic tumor inoculation and comparison of photon flux among three groups after the injection of luciferin. **(E)** Bioluminescent imaging from the same batch using one piece of cell sheet 2 weeks post implantation and photon flux data from eight formed tumor lesions.

The cell sheet transplantation method exhibited 100% tumorigenesis rate, which was higher than that in the suspension injection group (66.2 ± 1.058%, *p* < 0.0001) (Figure 2A). From the mass volume monitoring curve results, the cell sheet group’s volume increased faster than the suspension group. In addition, the two-piece cell sheet group mass increased more than the one-piece group (Figure 2B). The mass was observed as early as 10 days in the one-piece cell sheet transplantation group, and 7 days in two-piece cell sheet group (Supplementary Figure 2). On the other hand, it took 14 days or longer time to form a visible tumor mass in the suspension injection group. The weight curve of the mice tumor indicated that the cell sheet group exhibited more weight gain than the suspension injection group due to rapid tumor growth. However, the weight gain diminished 30 days after transplantation, due to starvation and the cachexia resulting from the heavy tumor load (Figure 2C). Apart from the high success rate in the cell sheet group, when transplanted with the two pieces of the cell sheet, the resultant tumor had higher bioactivity and tumor growth rate (Figure 2D). To evaluate the consistency of tumorigenesis using the cell sheet transplantation method, we performed the one-piece cell sheet implantation in eight sites of four mice. The results revealed 100% of successful tumor lesion formation and high consistency according to the photon flux analysis data (Figure 2E).

### MicroCT and Bone Destruction Analysis

**FIGURE 3:**
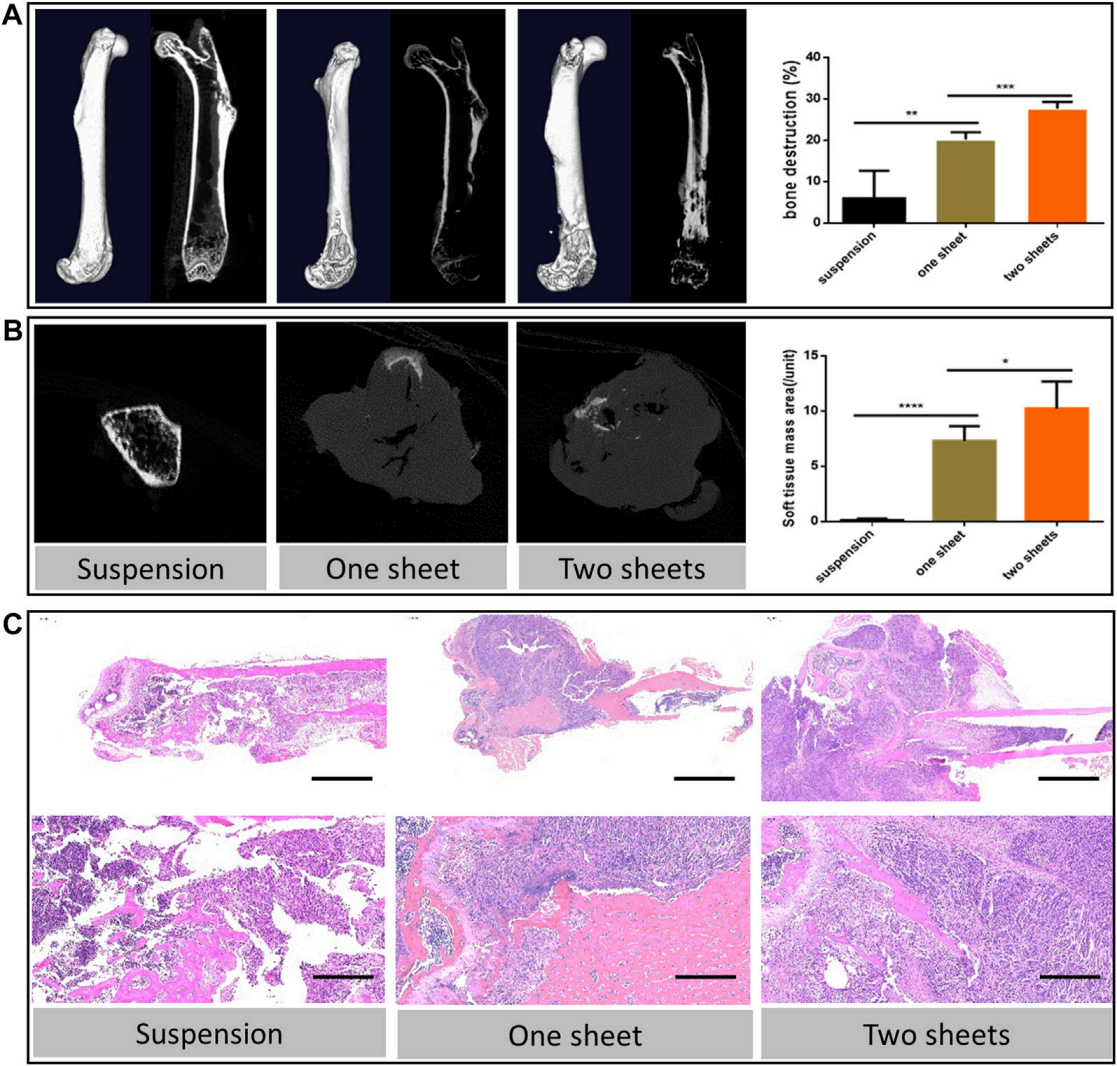
Bone destruction characterization **(A)** 3D reconstruction and coronal images of micro-CT scan from representative femur lesions and corresponding statistics analysis of bone destruction percentage. **(B)** Transverse images of the representative soft tissue mass and corresponding statistics analysis. **(C)** Hematoxylin and eosin staining of the representative bone lesion. Scale bar in upper panel = 2 mm; scale bar in lower panel = 200 μm.

MicroCT images indicated that the tumor cell caused bone destruction and the soft tissue mass at the inoculation site of the femur. From 3D reconstruction and the vertical view, bone destruction was observable in the whole cell sheet transplantation group, due to the 100% tumorigenesis rate. While in the suspension injection group, bone destruction varied in the tumor formation extent. With regard to the sample with no detectable tumor mass, bone destruction was confined to the bone cavity or scattered around the cortical bone. For the visible mass sample, the bone destruction level was similar to that of the cell sheet group. The result further revealed that both cell sheet transplantation groups had a higher bone destruction percentage (20.22 ± 1.566 and 27.70 ± 1.779) than the suspension injection group (5.988 ± 6.673, *p* < 0.01, Figure 3A). Besides, the destruction percentage of the two-piece cell sheet group (27.70 ± 1.779) was higher than that of the one-piece cell sheet group (20.22 ± 1.566, *p* < 0.001). Apart from the osteolysis change, there were ossification lesions in the two-cell sheet group. From the cross-section view results, the area of the soft tissue mass in the cell sheet transplantation group (7.300 ± 1.351 and 10.26 ± 2.437) was much higher than that in the suspension injection group (0.120 ± 0.1304) (*p* < 0.0001, Figure 3B).

### Histology Analysis

**FIGURE 4:**
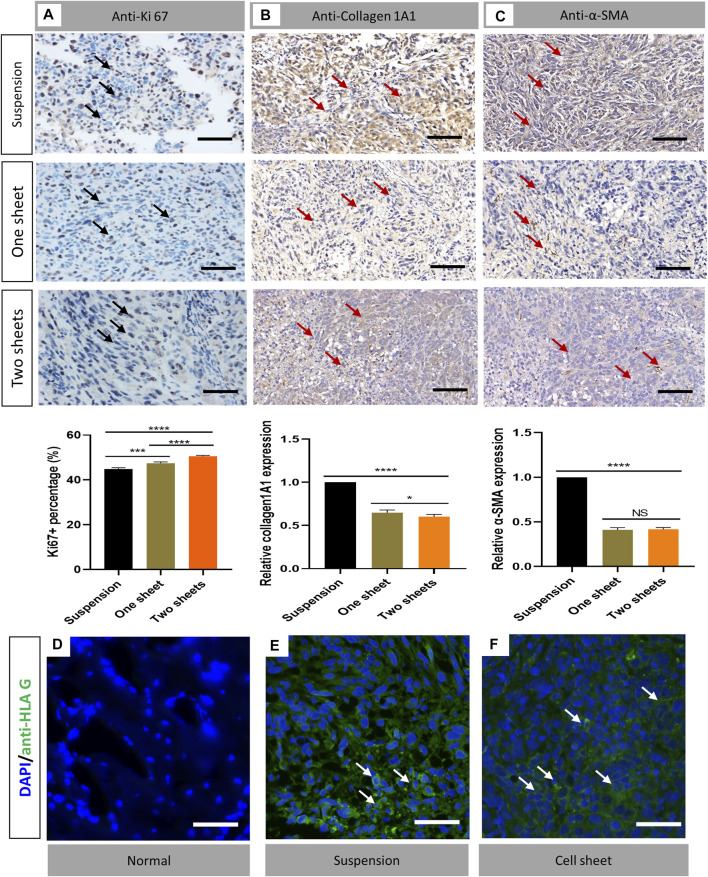
Immunostaining analysis of cell proliferation and detection of fibroblasts and cell origin. **(A)** Ki67 staining results of transverse sections of tumor in each group. **(B)** Collagen 1A1 staining of representative sample from each group **(C)**. α-SMA staining of representative sample from each group (n = 6). Statistical significance was calculated by Student’s t test. ^ns^p > 0.05, *p < 0.05, ***p < 0.001, ****p < 0.0001. Scale bar = 200 μm **(D–F)** Immunofluorescent staining of anti-HLA G (green) in normal bone, cell suspension–induced tumor and cell sheet–induced tumor samples, respectively. Scale bar = 50 μm.

The histological sections revealed typical pathological changes in various experimental groups (Figure 3C). To compare the difference between the three groups, we chose typical sections in each group for the assessment. Generally, tumor lesions and bone destruction were observed in all the groups. In the cell sheet group, there was no clear border between the normal and tumor tissue. Also, we observed that the trabecular bone was surrounded by the tumor tissue, suggesting that the tumor cells could cause osteolysis of the normal bone tissue. The results of Ki67 staining indicated there was a significant increase in the Ki67 expression in the cell sheet transplantation group (*p* < 0.0001), which revealed that the proliferation activity of tumor cells in the cell sheet transplantation group was increased (Figure 4A). To study the fibroblast biomarker expression in the tumor tissue, collagen 1A1 and α-SMA were stained. From the statistics results, the relevant expression of both the biomarkers in the cell sheet transplantation group was significantly lower than that in the suspension injection group (*p* < 0.0001), indicating the number of fibroblasts was higher in the suspension group than in the cell sheet group. Furthermore, there was only minor (Collagen 1A1, *p* < 0.05) or no significant (α-SMA) difference between one sheet and two sheets group (Figures 4B,C), indicating that fibroblast infiltration was similar in cell sheet–induced tumor. Anti-HLA G immunofluorescence data demonstrated that the tumor cells originated from the human cells (Figures 4E,F) by comparing with the normal mice femur sample (Figure 4D).

### Drug Test Analysis and Tumor Apoptosis Investigation

**FIGURE 5:**
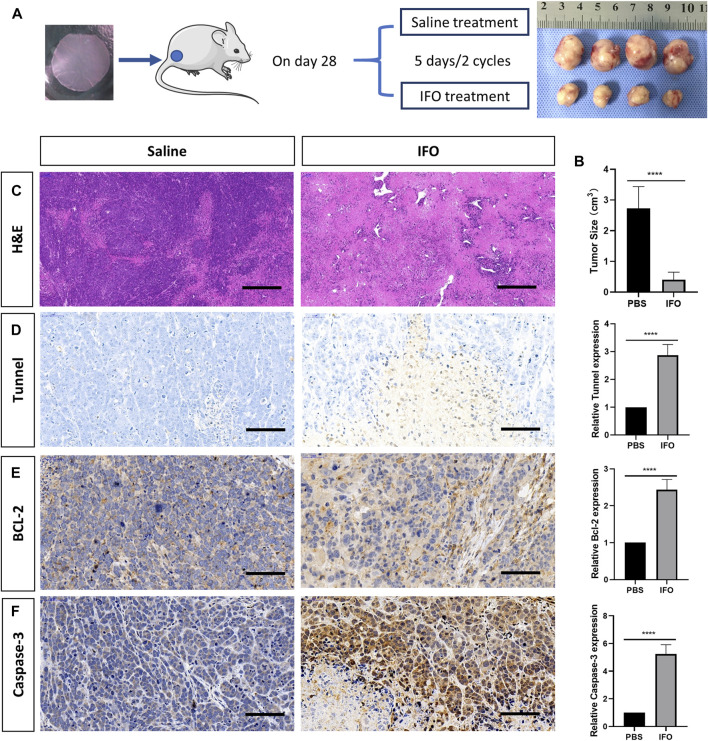
Apoptosis analysis of tumor after treatment of ifosfamide (IFO). **(A)** Schematic graph of the treatment plan and the result tumor sample after treatment. **(B)** Hematoxylin and eosin staining of the representative bone lesion from saline and IFO treatment group, respectively. **(C)** Tumor size after the treatment in each group **(D)** TUNEL staining of the representative bone lesion from saline and IFO treatment group, respectively. **(E)** BCL-2 staining of the representative bone lesion from saline and IFO treatment group, respectively. **(F)** Caspase-3 staining of the representative bone lesion from PBS and IFO treatment group, respectively. Statistical significance was calculated by Student’s t test. ****p < 0.0001. Scale bar = 200 μm.

All mice survived after two cycles of IFO treatment. Our data showed that the tumor size was 2.728 ± 0.323 cm^3^ in the IFO-treated group and 0.405 ± 0.102 cm^3^ in the control group at the end of the drug administration. The result indicated the tumor growth was significantly inhibited by IFO treatment (*p* < 0.001, Figure 5B). Compared to the control group, our data also revealed that the relevant expression level of TUNEL, Bcl-2, and caspase-3 in IFO group elevated 1.87 ± 0.16, 1.43 ± 0.11, and 4.23 ± 0.27, respectively (*p* < 0.0001, Figures 5C–E), by comparing with the control group. These data indicated that the tumor proliferation rate was significantly inhibited by the antitumor drug, and the result in each mouse was consistent.

## Discussion

This study introduced an efficient orthotopic osteosarcoma model in mice. The model is anticipated to reduce the animal use and could be used extensively in the laboratory. We realized that it took as less as 7 days to form a visible mass in nude mice in our model with 100% tumorigenesis rate, whereas a similar amount of cell suspension injection took more than 15 days with less than 67% tumorigenesis rate. The microCT results indicated a large extent of bone destruction and soft tissue mass formation. This suggested typical osteosarcoma symptoms like tumor bone growth and bone destruction. This approach could significantly reduce animal use in the experiment and expedite the research cycle, which could also reduce the research expense. Furthermore, this typical model had been proved to be an efficient tool for drug test, which is beneficial to antitumor screening.

Basically, the cell sheet method has obvious advantage over the cell suspension method; this was proved in other previous studies (Suzuki et al., 2014). Akimoto transplanted hepatocarcinoma cell sheet orthotopically and successfully to induce efficient intrahepatic tumor (Akimoto et al., 2019). He also compared the immunological reaction between immunodeficient mice and immunocompetent mice by comparing cell sheet transplantation and cell suspension injection with mouse lung squamous cancer cells (KLN-205) and found that the inoculated cells had a better survival rate and a lower immunological reaction in the cell sheet group (Akimoto et al., 2018). However, the success rate of tumor modeling in nude mice also depends on the cell type. Various transplanted cell lines impact the tumor formation and lung metastasis rates (Yuan et al., 2009; Sottnik et al., 2010). The malignant tumor cells secrete E-cadherin and matrix metalloproteinases (MMPs) (Jiang et al., 2015). The MMPs can degrade various protein components of the extracellular matrix (ECM). MMP hydrolysis results in loose connections between the cells, making it difficult for tumor cells to form tight junctions unlike normal cells. As such, tumor cells metastasize easily *in vivo*. However, our experimental findings confirmed that a high-density osteosarcoma cell culture formed a considerable amount of the extracellular matrix and complete cell sheets, and detached from the temperature-sensitive culture plate. This was consistent with the preparation methods of other temperature-sensitive cell tablets (Isenberg et al., 2008; Guo et al., 2015).

Our findings further demonstrated that tissue engineering for tumor inoculation and transplantation was feasible and effective. The cell sheets had a complete extracellular matrix and exhibited high cellular viability. Hence, it was difficult to lose the cells. Generally, the advantages of these new tumor models include rapid tumorigenesis and high consistency. As such, it can be used as a novel reference model by other researchers in establishing other mouse tumor models. This contradicts the findings reported in other articles using orthotopic xenotransplantation of human osteosarcoma cell lines (Igarashi et al., 2020). There are numerous techniques for constructing a mouse tumor model. The most commonly used materials are cell suspension and tissue mass. In establishing animal models of transplanted tumors, cell suspension is often used as grafts, although the experimental results are not ideal. In this mode, single cells lost the cell–cell junctions and ECM proteins after being digested. This may result into low adherence of single cells to the host tissue and a low survival rate. Some scholars suggest that the cells used for transplantation are acted on by enzymatic hydrolysis. Besides, the structure of tumor cells is destroyed, resulting in changes in the tumor biological behavior, which may in turn impact the proliferation and metastasis of human tumor cells following the transplantation (Fu et al., 1992). The novel technique we established does not require trypsin digestion, and this not only ensures tumorigenesis but also maintains the inherent characteristics and vitality of tumor cells at a relatively lower cost.

In this study, unlike other 3D culture system, the cell sheet exhibited the same proliferation rate with the traditional 2D culture. However, the *in vivo* cell sheet–induced tumor displayed a higher proliferation rate than the suspension injection method. This is in line with other studies (Akimoto Jun et al., 2018; Akimoto Jun et al., 2019). We consider there were several reasons. First, the viability of suspension cells decreased after trypsin digestion. Second, although with the same amount of the initial inoculation cell number, there were more cells lost in the suspension group than in the cell sheet group. Third, the host tissue may affect the proliferation of scatted tumor cells. Therefore, we stained the fibroblast biomarkers to investigate the difference of fibroblast infiltration between the suspension group and the cell sheet group. The results revealed fibroblast expression in the suspension group was higher than that in the cell sheet group. That implied existence of fibroblast may impact the tumor cell proliferation. Previous studies pointed out that tumor-associated fibroblasts may promote tumor development and metastasis formation (Bhowmick et al., 2004; Mishra et al., 2011; Calon et al., 2014). However, other scholars reported that cancer-associated fibroblasts (CAFs) have both tumor-promoting and tumor-suppressive functions (Park et al., 2020). Hence, we speculated that the tumor growth might be enhanced by the inherent tumor-associated fibroblasts, rather than the interstitial fibroblasts. A biomimic osteosarcoma cell constructs might be helpful to realize this hypothesis.

Last, we performed the drug test in this tumor model by administrating IFO. The IFO is the most common and first-line antitumor drug in treating osteosarcoma (Goorin et al., 2002; Palmerini et al., 2020). Although it has side effects such as renal damage, mesna could be a safe and effective agent to reduce the toxicity (Marti et al., 1985). IFO could delay the cell cycle at the G stage and influence cross-linking with DNA (Zhang et al., 2018). The drug test results in this study reflected the expected outcome of the antitumor treatment. In the IFO-treated group, the size of the tumor diminished significantly by comparing with the saline-treated group, as expected. As a result, the H&E staining in the IFO group displayed extensive cell necrosis and apoptosis. However, few studies were conducted on the histology changes after IFO therapy in recent years (Heymann et al., 2005). On the contrary, plenty of *in vitro* studies have proved that IFO inhibits tumor growth by inducing cancer cell apoptosis such as the TUNEL and Bcl-2 expression elevation (Chen et al., 2015; Wang et al., 2018). Therefore, our study further demonstrated that a significantly higher expression level of TUNEL, Bcl-2, and caspase-3 was observed in the IFO-treated group. This was consistent with those previous *in vitro* studies.

This work aims to establish an osteosarcoma mouse model that simulates the patient’s disease pattern more accurately than the subcutaneous injection of tumor cells into rodents. The advantages associated with the model are as follows: fast tumor formation in mice, which greatly lowers the number of animals required for conducting the experiment; and the rapid progression of the disease means that research can be performed within a short period to minimize the impact on animals. This not only satisfactorily simulated the occurrence and development of osteosarcoma but also suggested that we can control the number of cell sheets to meet the requirements of modeling, for instance, tumor formation time and size. In comparison to the immune normal mouse model, immunodeficient mice can also be used to study the immune microenvironment of tumors and the potential immunotherapy. We also compared the probability of lung metastasis between cell suspension injection and cell sheet transplantation. The findings showed that there was a difference in the probability of lung metastasis between suspension injection and cell sheet transplantation, suggesting that they represented two distinct cell invasive behaviors and tumor microenvironments.

The shortcomings of our current method included the success rate of cell sheet culture was not high (50–60%); in comparison to the traditional cell suspension injection method, the cell culture cost of this method is higher, and it is possible that the cell sheets cannot be concentrated in the transplant site. Judging from the existing experimental results, although the tumor formation rate is 100%, there is still a situation whereby the tumor size is not consistent. And it was not always easy to transfer the intact cell sheet without rupture. Although with the help of a syringe, this shortcoming could be diminished, the accompanied excess medium would affect the cell sheet localization. Therefore, methods could be further improved in the future.

## Conclusion

A novel method of constructing a tumor animal model was established in this study to explore the effect of cell sheets on tumor formation in mice. The newly established animal model not only lays a foundation for further studies on the biological function of osteosarcoma in the laboratory but also provides a reference for the future construction of other tumor animal models. The study emphasizes the importance of small-scale pilot studies before obtaining the experimental data, to allow for improved techniques and accurate sample size calculations. In addition, it increases the probability of obtaining statistically meaningful data while minimizing the number of experimental animals.

## Data Availability

The original contributions presented in the study are included in the article/Supplementary Material; further inquiries can be directed to the corresponding authors.
