# Supplementary figures and images for: Efficient and Consistent Orthotopic Osteosarcoma Model by Cell Sheet Transplantation in the Nude Mice for Drug Testing
Source: Front Bioeng Biotechnol. 2021 Sep 24;9:690409. doi: 10.3389/fbioe.2021.690409 (PMC8498338; doi:10.3389/fbioe.2021.690409)

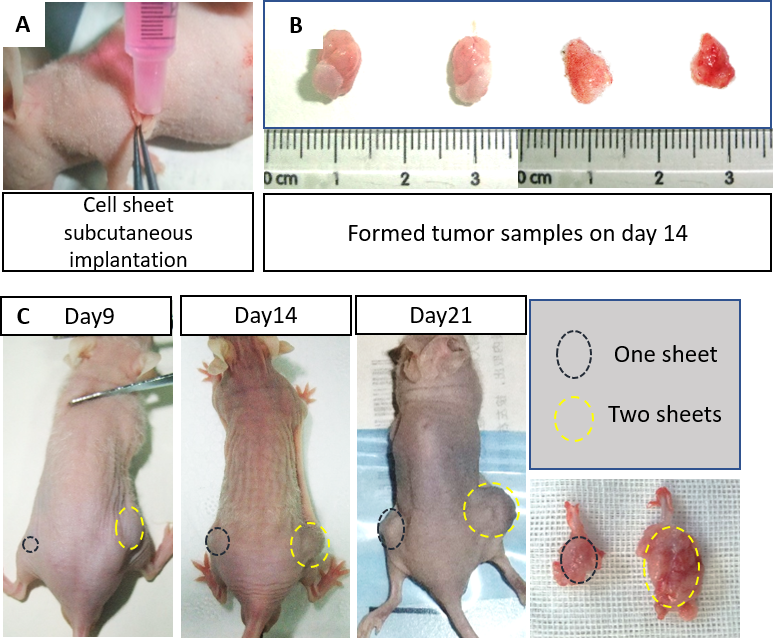

Supplement: Supplementary file 1 [file Image2.PNG]

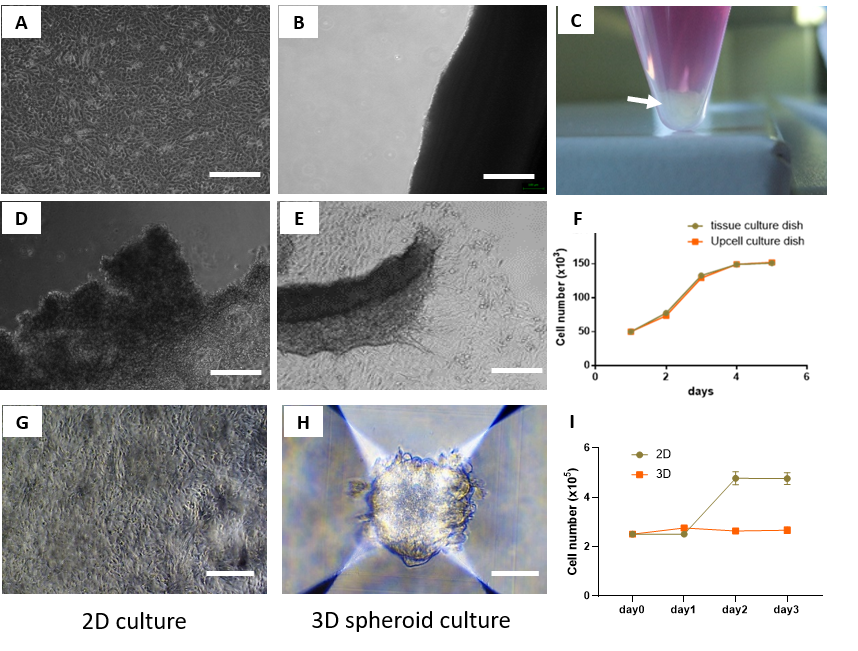

Supplement: Supplementary file 2 [file Image1.PNG]
